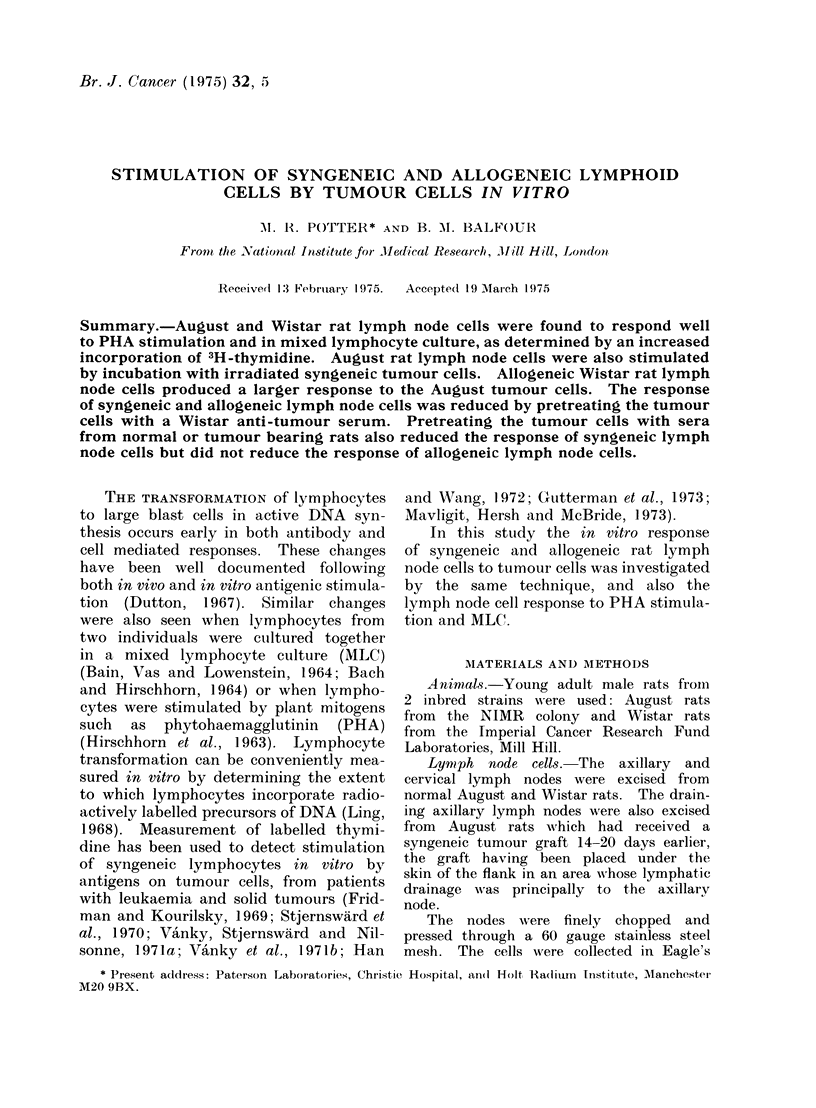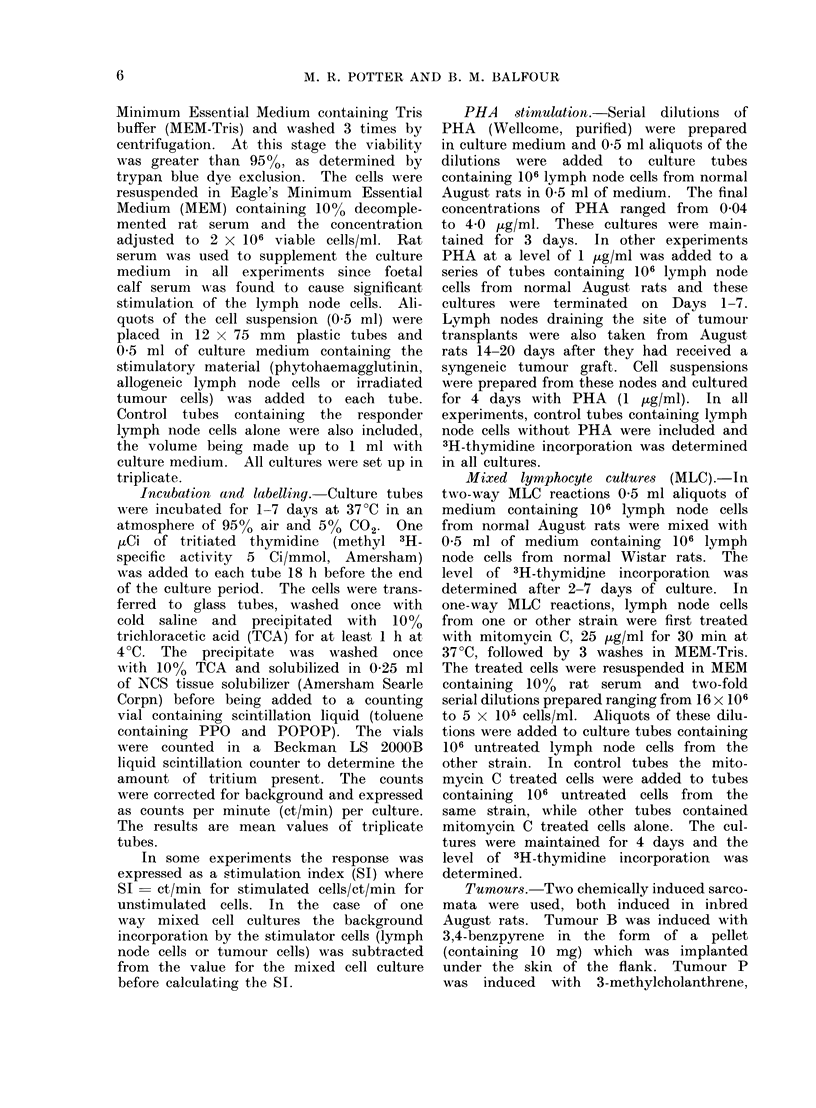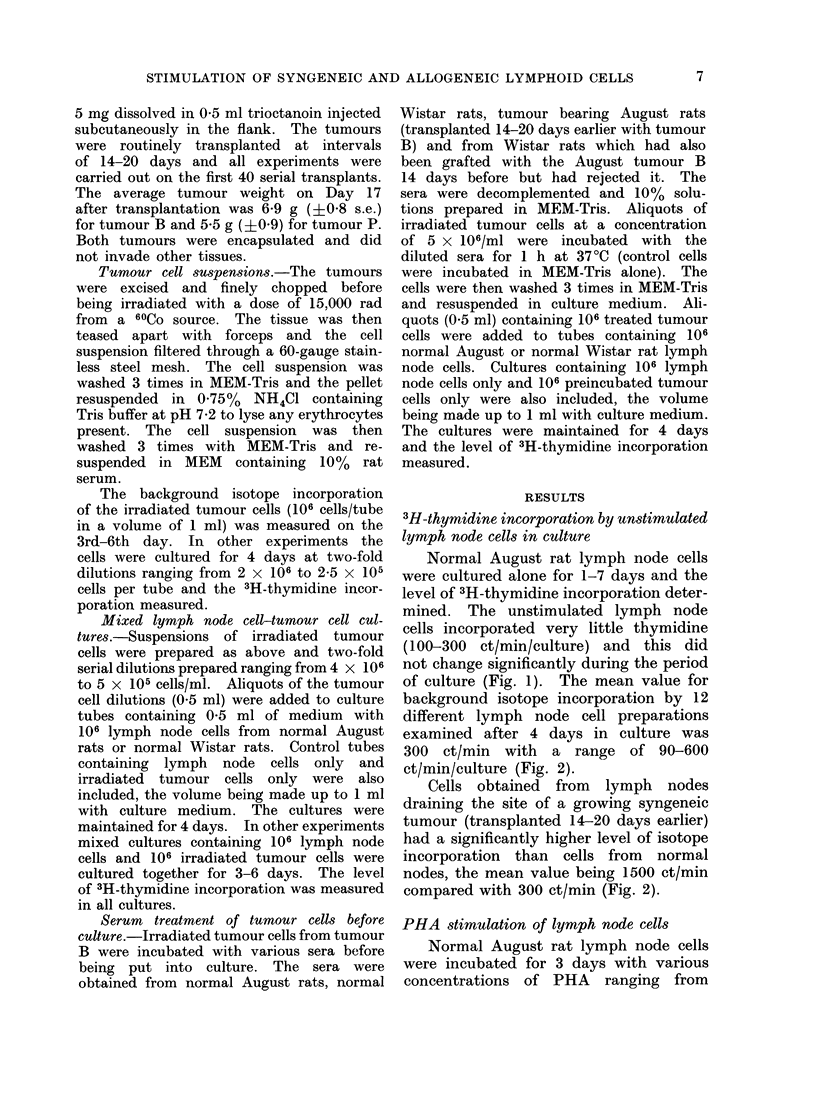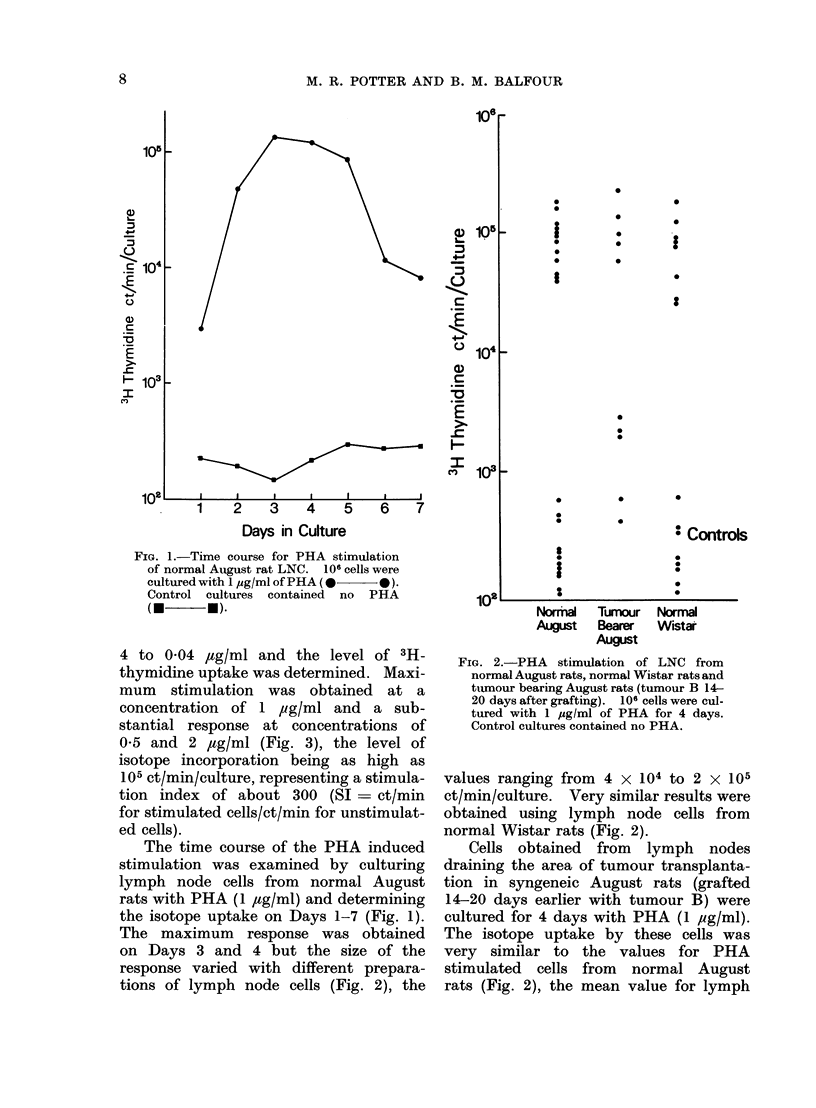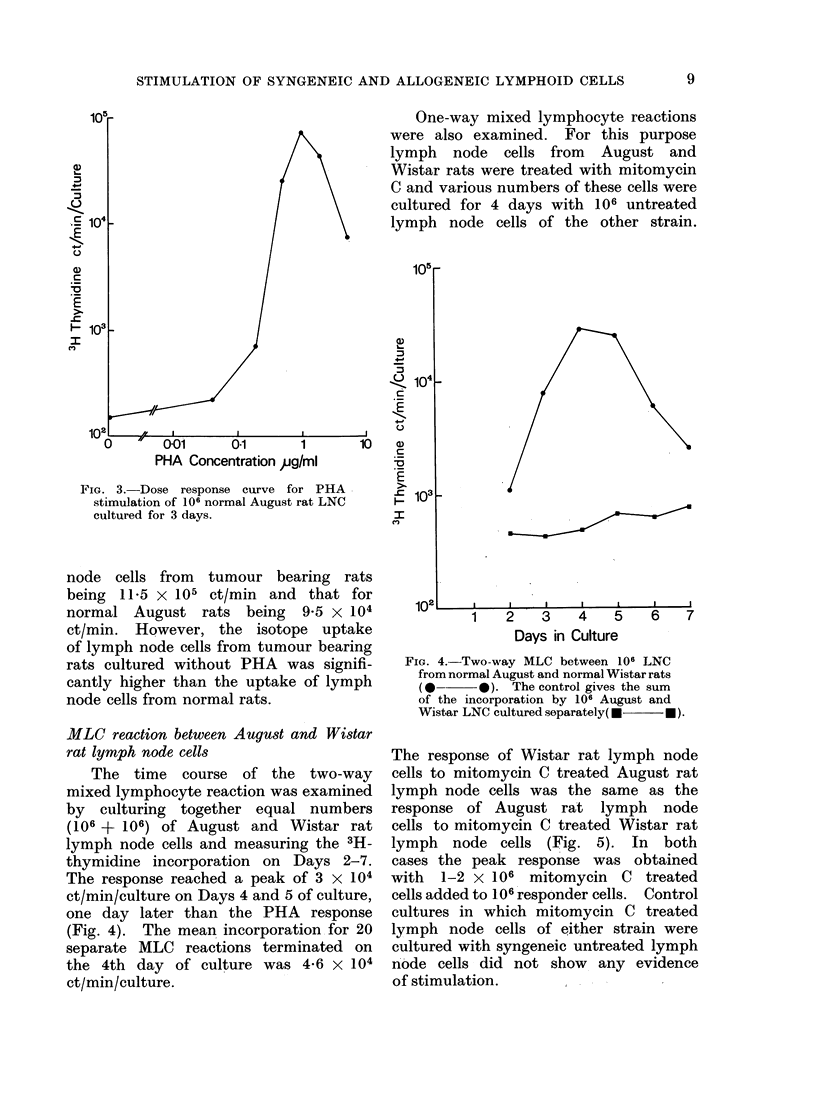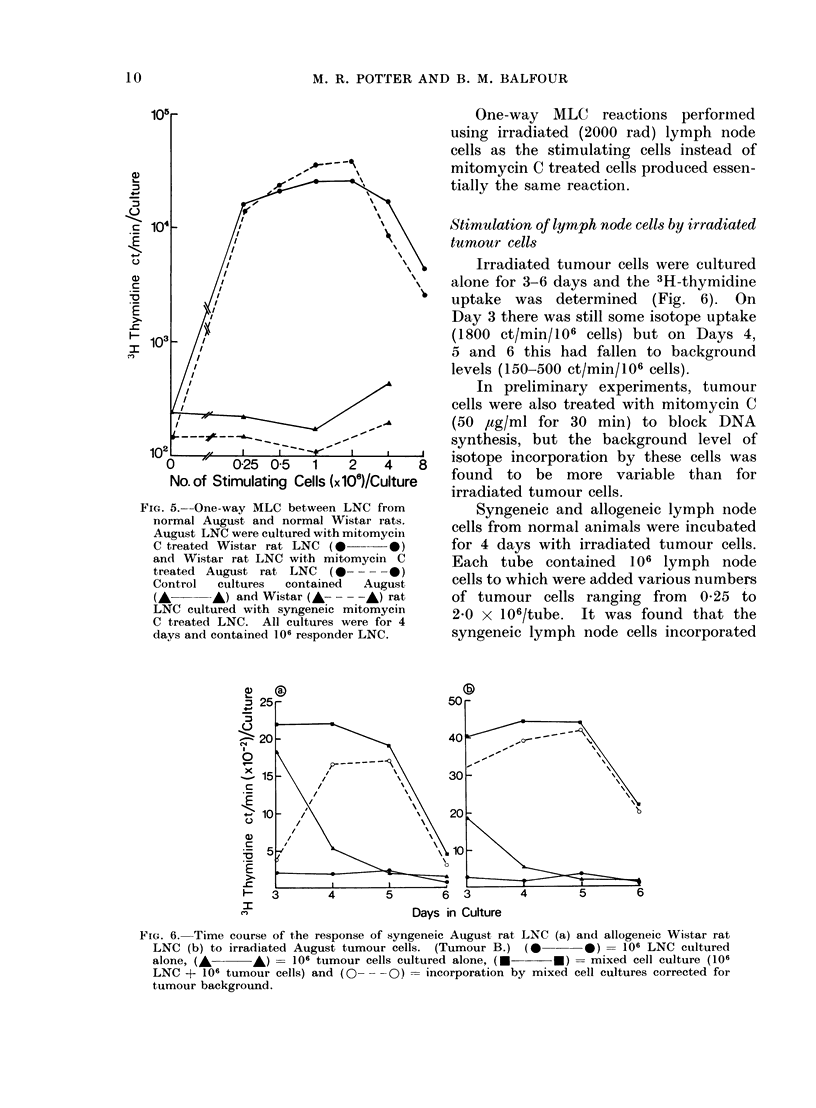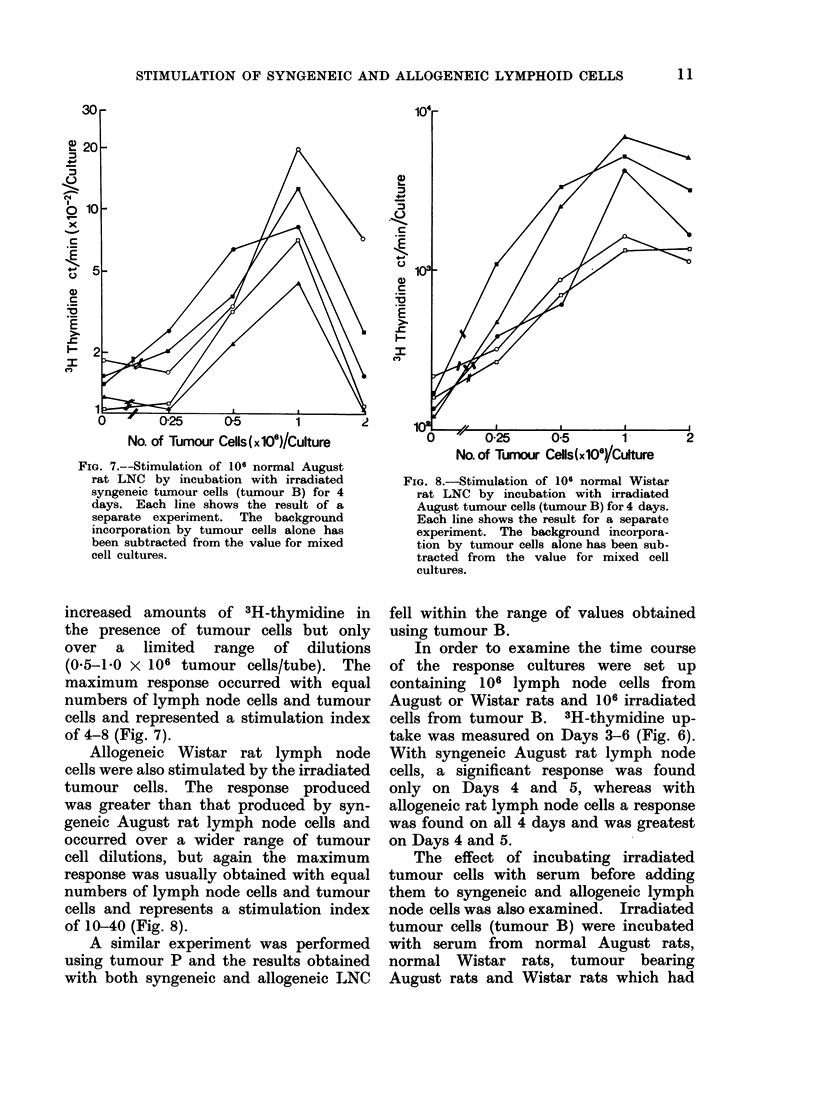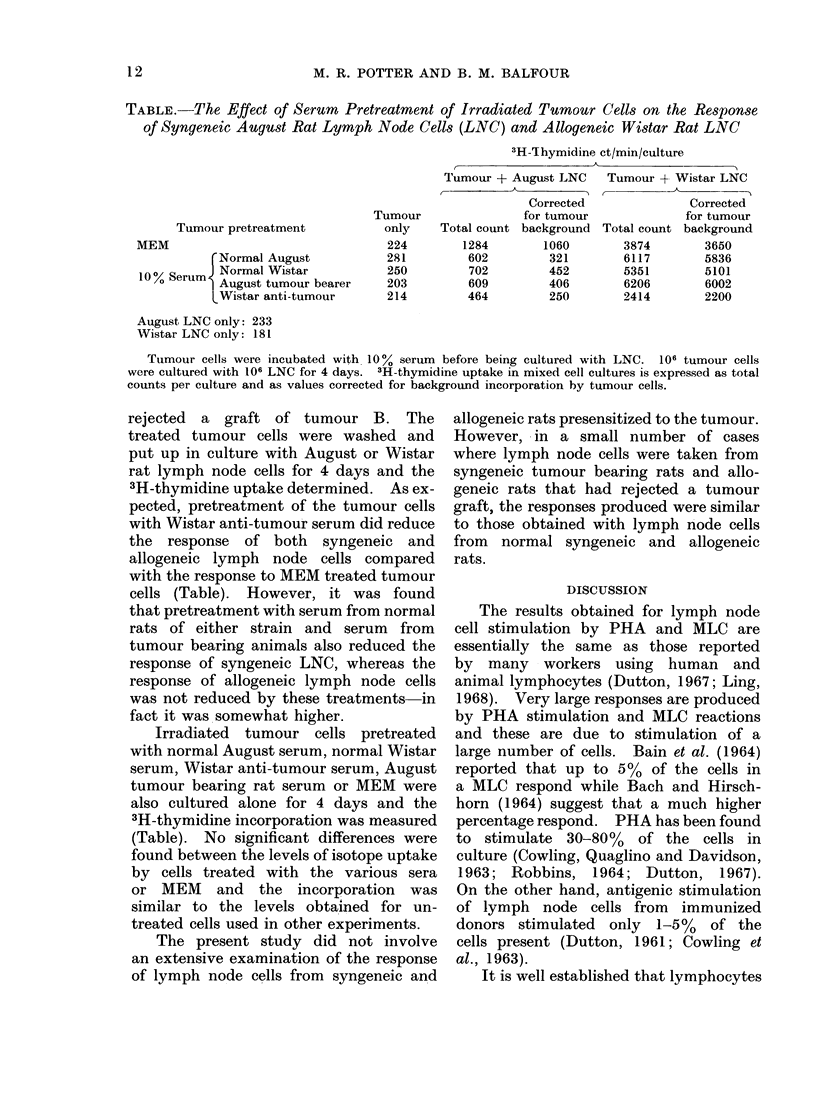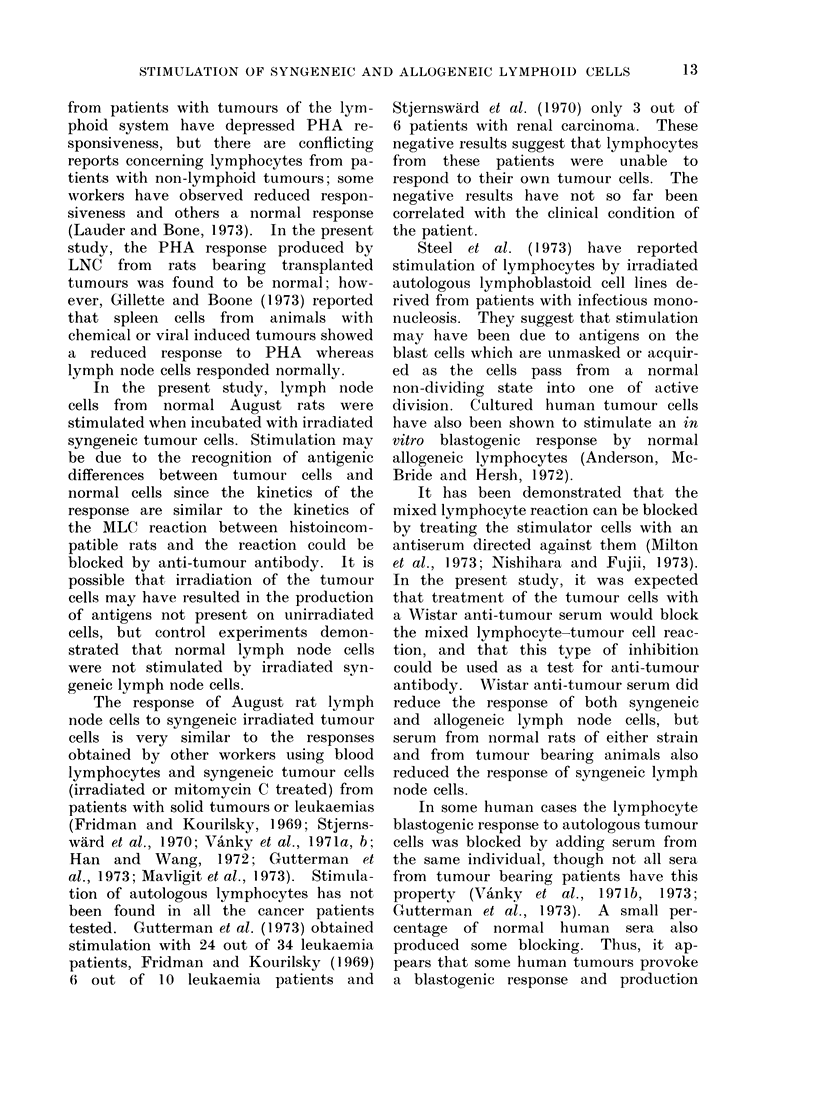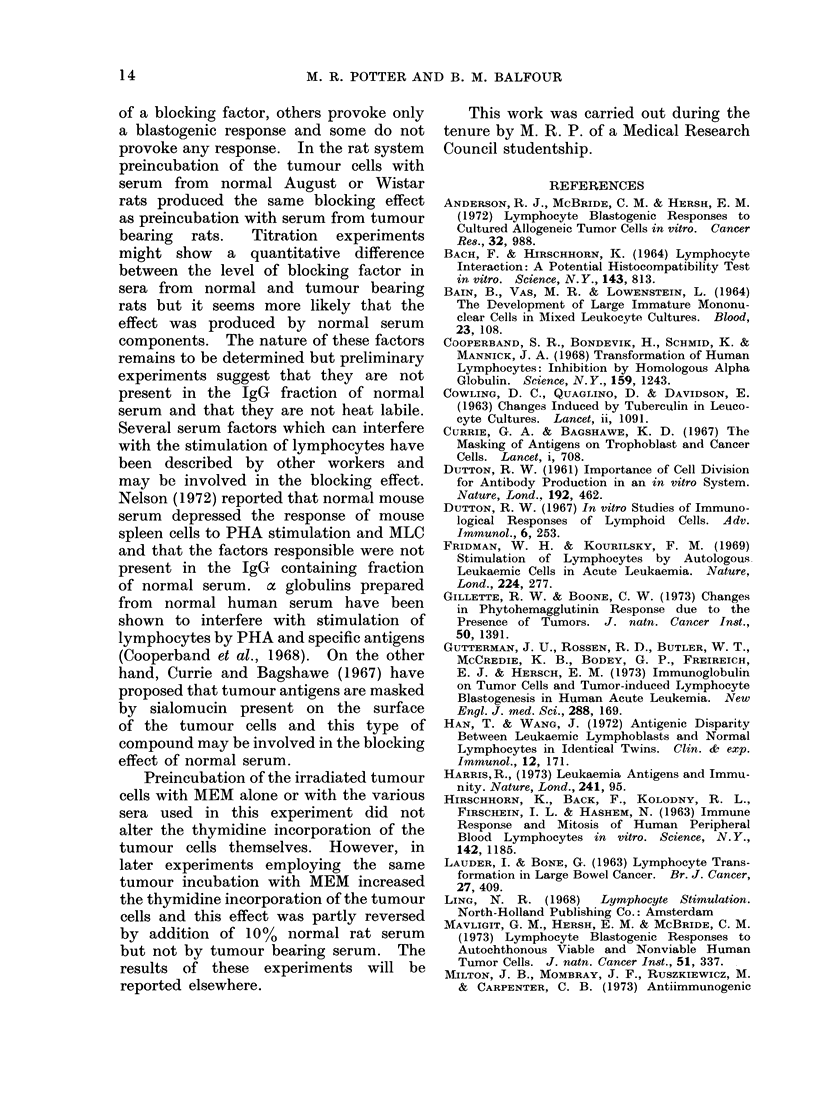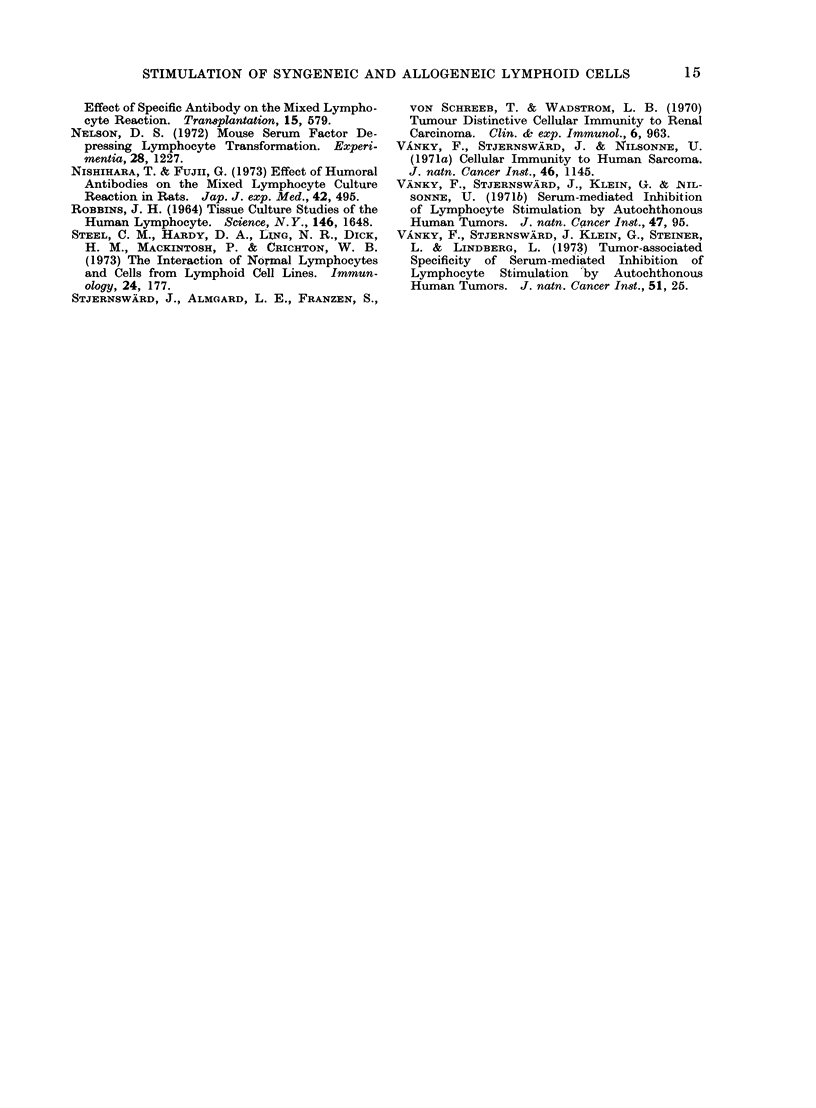# Stimulation of syngeneic and allogeneic lymphoid cells by tumour cells in vitro.

**DOI:** 10.1038/bjc.1975.127

**Published:** 1975-07

**Authors:** M. R. Potter, B. M. Balfour

## Abstract

August and Wistar rat lymph node cells were found to respond well to PHA stimulation and in mixed lymphocyte culture, as determined by an increased incorporation of 3H-thymidine. August rat lymph node cells were also stimulated by incubation with irradiated syngeneic tumour cells. Allogeneic Wistar rat lymph node cells produced a larger response to the August tumour cells. The response of syngeneic and allogeneic lymph node cells was reduced by pretreating the tumour cells with a Wistar anti-tumour serum. Pretreating the tumour cells with sera from normal or tumour bearing rats also reduced the response of syngeneic lymph node cells but did not reduce the response of allogeneic lymph node cells.


*Br. J. Cancer* (1975) **32**, 5

### STIMULATION OF SYNGENEIC AND ALLOGENEIC LYMPHOID CELLS BY TUMOUR CELLS *IN VITRO*

M. R. POTTER* AND B. M. BALFOUR

*From the National Institute for Medical Research, Mill Hill, London*

Received 13 February 1975. Accepted 19 March 1975

**Summary.—August and Wistar rat lymph node cells were found to respond well to PHA stimulation and in mixed lymphocyte culture, as determined by an increased incorporation of $^3$H-thymidine. August rat lymph node cells were also stimulated by incubation with irradiated syngeneic tumour cells. Allogeneic Wistar rat lymph node cells produced a larger response to the August tumour cells. The response of syngeneic and allogeneic lymph node cells was reduced by pretreating the tumour cells with a Wistar anti-tumour serum. Pretreating the tumour cells with sera from normal or tumour bearing rats also reduced the response of syngeneic lymph node cells but did not reduce the response of allogeneic lymph node cells.**

THE TRANSFORMATION of lymphocytes to large blast cells in active DNA synthesis occurs early in both antibody and cell mediated responses. These changes have been well documented following both *in vivo* and *in vitro* antigenic stimulation (Dutton, 1967). Similar changes were also seen when lymphocytes from two individuals were cultured together in a mixed lymphocyte culture (MLC) (Bain, Vas and Lowenstein, 1964; Bach and Hirschhorn, 1964) or when lymphocytes were stimulated by plant mitogens such as phytohaemagglutinin (PHA) (Hirschhorn *et al.*, 1963). Lymphocyte transformation can be conveniently measured *in vitro* by determining the extent to which lymphocytes incorporate radioactively labelled precursors of DNA (Ling, 1968). Measurement of labelled thymidine has been used to detect stimulation of syngeneic lymphocytes *in vitro* by antigens on tumour cells, from patients with leukaemia and solid tumours (Fridman and Kourilsky, 1969; Stjernswärd *et al.*, 1970; Vánky, Stjernswärd and Nilsonne, 1971*a*; Vánky *et al.*, 1971*b*; Han and Wang, 1972; Gutterman *et al.*, 1973; Mavligit, Hersh and McBride, 1973).

In this study the *in vitro* response of syngeneic and allogeneic rat lymph node cells to tumour cells was investigated by the same technique, and also the lymph node cell response to PHA stimulation and MLC.

### MATERIALS AND METHODS

*Animals.*—Young adult male rats from 2 inbred strains were used: August rats from the NIMR colony and Wistar rats from the Imperial Cancer Research Fund Laboratories, Mill Hill.

*Lymph node cells.*—The axillary and cervical lymph nodes were excised from normal August and Wistar rats. The draining axillary lymph nodes were also excised from August rats which had received a syngeneic tumour graft 14–20 days earlier, the graft having been placed under the skin of the flank in an area whose lymphatic drainage was principally to the axillary node.

The nodes were finely chopped and pressed through a 60 gauge stainless steel mesh. The cells were collected in Eagle's

* Present address: Paterson Laboratories, Christie Hospital, and Holt Radium Institute, Manchester M20 9BX.

Minimum Essential Medium containing Tris buffer (MEM-Tris) and washed 3 times by centrifugation. At this stage the viability was greater than 95%, as determined by trypan blue dye exclusion. The cells were resuspended in Eagle's Minimum Essential Medium (MEM) containing 10% decomplemented rat serum and the concentration adjusted to $2 \times 10^6$ viable cells/ml. Rat serum was used to supplement the culture medium in all experiments since foetal calf serum was found to cause significant stimulation of the lymph node cells. Aliquots of the cell suspension (0·5 ml) were placed in 12 × 75 mm plastic tubes and 0·5 ml of culture medium containing the stimulatory material (phytohaemagglutinin, allogeneic lymph node cells or irradiated tumour cells) was added to each tube. Control tubes containing the responder lymph node cells alone were also included, the volume being made up to 1 ml with culture medium. All cultures were set up in triplicate.

*Incubation and labelling.*—Culture tubes were incubated for 1–7 days at 37°C in an atmosphere of 95% air and 5% $CO_2$. One $\mu$Ci of tritiated thymidine (methyl $^3$H-specific activity 5 Ci/mmol, Amersham) was added to each tube 18 h before the end of the culture period. The cells were transferred to glass tubes, washed once with cold saline and precipitated with 10% trichloracetic acid (TCA) for at least 1 h at 4°C. The precipitate was washed once with 10% TCA and solubilized in 0·25 ml of NCS tissue solubilizer (Amersham Searle Corpn) before being added to a counting vial containing scintillation liquid (toluene containing PPO and POPOP). The vials were counted in a Beckman LS 2000B liquid scintillation counter to determine the amount of tritium present. The counts were corrected for background and expressed as counts per minute (ct/min) per culture. The results are mean values of triplicate tubes.

In some experiments the response was expressed as a stimulation index (SI) where SI = ct/min for stimulated cells/ct/min for unstimulated cells. In the case of one way mixed cell cultures the background incorporation by the stimulator cells (lymph node cells or tumour cells) was subtracted from the value for the mixed cell culture before calculating the SI.

*PHA stimulation.*—Serial dilutions of PHA (Wellcome, purified) were prepared in culture medium and 0·5 ml aliquots of the dilutions were added to culture tubes containing $10^6$ lymph node cells from normal August rats in 0·5 ml of medium. The final concentrations of PHA ranged from 0·04 to 4·0 $\mu$g/ml. These cultures were maintained for 3 days. In other experiments PHA at a level of 1 $\mu$g/ml was added to a series of tubes containing $10^6$ lymph node cells from normal August rats and these cultures were terminated on Days 1–7. Lymph nodes draining the site of tumour transplants were also taken from August rats 14–20 days after they had received a syngeneic tumour graft. Cell suspensions were prepared from these nodes and cultured for 4 days with PHA (1 $\mu$g/ml). In all experiments, control tubes containing lymph node cells without PHA were included and $^3$H-thymidine incorporation was determined in all cultures.

*Mixed lymphocyte cultures* (MLC).—In two-way MLC reactions 0·5 ml aliquots of medium containing $10^6$ lymph node cells from normal August rats were mixed with 0·5 ml of medium containing $10^6$ lymph node cells from normal Wistar rats. The level of $^3$H-thymidine incorporation was determined after 2–7 days of culture. In one-way MLC reactions, lymph node cells from one or other strain were first treated with mitomycin C, 25 $\mu$g/ml for 30 min at 37°C, followed by 3 washes in MEM-Tris. The treated cells were resuspended in MEM containing 10% rat serum and two-fold serial dilutions prepared ranging from $16 \times 10^6$ to $5 \times 10^5$ cells/ml. Aliquots of these dilutions were added to culture tubes containing $10^6$ untreated lymph node cells from the other strain. In control tubes the mitomycin C treated cells were added to tubes containing $10^6$ untreated cells from the same strain, while other tubes contained mitomycin C treated cells alone. The cultures were maintained for 4 days and the level of $^3$H-thymidine incorporation was determined.

*Tumours.*—Two chemically induced sarcomata were used, both induced in inbred August rats. Tumour B was induced with 3,4-benzpyrene in the form of a pellet (containing 10 mg) which was implanted under the skin of the flank. Tumour P was induced with 3-methylcholanthrene,

5 mg dissolved in 0·5 ml trioctanoin injected subcutaneously in the flank. The tumours were routinely transplanted at intervals of 14–20 days and all experiments were carried out on the first 40 serial transplants. The average tumour weight on Day 17 after transplantation was 6·9 g ($\pm$0·8 s.e.) for tumour B and 5·5 g ($\pm$0·9) for tumour P. Both tumours were encapsulated and did not invade other tissues.

*Tumour cell suspensions.*—The tumours were excised and finely chopped before being irradiated with a dose of 15,000 rad from a $^{60}$Co source. The tissue was then teased apart with forceps and the cell suspension filtered through a 60-gauge stainless steel mesh. The cell suspension was washed 3 times in MEM-Tris and the pellet resuspended in 0·75% $NH_4Cl$ containing Tris buffer at pH 7·2 to lyse any erythrocytes present. The cell suspension was then washed 3 times with MEM-Tris and resuspended in MEM containing 10% rat serum.

The background isotope incorporation of the irradiated tumour cells ($10^6$ cells/tube in a volume of 1 ml) was measured on the 3rd–6th day. In other experiments the cells were cultured for 4 days at two-fold dilutions ranging from $2 \times 10^6$ to $2{\cdot}5 \times 10^5$ cells per tube and the $^3$H-thymidine incorporation measured.

*Mixed lymph node cell–tumour cell cultures.*—Suspensions of irradiated tumour cells were prepared as above and two-fold serial dilutions prepared ranging from $4 \times 10^6$ to $5 \times 10^5$ cells/ml. Aliquots of the tumour cell dilutions (0·5 ml) were added to culture tubes containing 0·5 ml of medium with $10^6$ lymph node cells from normal August rats or normal Wistar rats. Control tubes containing lymph node cells only and irradiated tumour cells only were also included, the volume being made up to 1 ml with culture medium. The cultures were maintained for 4 days. In other experiments mixed cultures containing $10^6$ lymph node cells and $10^6$ irradiated tumour cells were cultured together for 3–6 days. The level of $^3$H-thymidine incorporation was measured in all cultures.

*Serum treatment of tumour cells before culture.*—Irradiated tumour cells from tumour B were incubated with various sera before being put into culture. The sera were obtained from normal August rats, normal Wistar rats, tumour bearing August rats (transplanted 14–20 days earlier with tumour B) and from Wistar rats which had also been grafted with the August tumour B 14 days before but had rejected it. The sera were decomplemented and 10% solutions prepared in MEM-Tris. Aliquots of irradiated tumour cells at a concentration of $5 \times 10^6$/ml were incubated with the diluted sera for 1 h at 37°C (control cells were incubated in MEM-Tris alone). The cells were then washed 3 times in MEM-Tris and resuspended in culture medium. Aliquots (0·5 ml) containing $10^6$ treated tumour cells were added to tubes containing $10^6$ normal August or normal Wistar rat lymph node cells. Cultures containing $10^6$ lymph node cells only and $10^6$ preincubated tumour cells only were also included, the volume being made up to 1 ml with culture medium. The cultures were maintained for 4 days and the level of $^3$H-thymidine incorporation measured.

### RESULTS

### *$^3$H-thymidine incorporation by unstimulated lymph node cells in culture*

Normal August rat lymph node cells were cultured alone for 1–7 days and the level of $^3$H-thymidine incorporation determined. The unstimulated lymph node cells incorporated very little thymidine (100–300 ct/min/culture) and this did not change significantly during the period of culture (Fig. 1). The mean value for background isotope incorporation by 12 different lymph node cell preparations examined after 4 days in culture was 300 ct/min with a range of 90–600 ct/min/culture (Fig. 2).

Cells obtained from lymph nodes draining the site of a growing syngeneic tumour (transplanted 14–20 days earlier) had a significantly higher level of isotope incorporation than cells from normal nodes, the mean value being 1500 ct/min compared with 300 ct/min (Fig. 2).

### *PHA stimulation of lymph node cells*

Normal August rat lymph node cells were incubated for 3 days with various concentrations of PHA ranging from

FIG. 1.—Time course for PHA stimulation of normal August rat LNC. $10^6$ cells were cultured with 1 $\mu$g/ml of PHA (●——●). Control cultures contained no PHA (■——■).

FIG. 2.—PHA stimulation of LNC from normal August rats, normal Wistar rats and tumour bearing August rats (tumour B 14–20 days after grafting). $10^6$ cells were cultured with 1 $\mu$g/ml of PHA for 4 days. Control cultures contained no PHA.

4 to 0·04 $\mu$g/ml and the level of $^3$H-thymidine uptake was determined. Maximum stimulation was obtained at a concentration of 1 $\mu$g/ml and a substantial response at concentrations of 0·5 and 2 $\mu$g/ml (Fig. 3), the level of isotope incorporation being as high as $10^5$ ct/min/culture, representing a stimulation index of about 300 (SI = ct/min for stimulated cells/ct/min for unstimulated cells).

The time course of the PHA induced stimulation was examined by culturing lymph node cells from normal August rats with PHA (1 $\mu$g/ml) and determining the isotope uptake on Days 1–7 (Fig. 1). The maximum response was obtained on Days 3 and 4 but the size of the response varied with different preparations of lymph node cells (Fig. 2), the values ranging from $4 \times 10^4$ to $2 \times 10^5$ ct/min/culture. Very similar results were obtained using lymph node cells from normal Wistar rats (Fig. 2).

Cells obtained from lymph nodes draining the area of tumour transplantation in syngeneic August rats (grafted 14–20 days earlier with tumour B) were cultured for 4 days with PHA (1 $\mu$g/ml). The isotope uptake by these cells was very similar to the values for PHA stimulated cells from normal August rats (Fig. 2), the mean value for lymph

FIG. 3.—Dose response curve for PHA stimulation of $10^6$ normal August rat LNC cultured for 3 days.

node cells from tumour bearing rats being $11 \cdot 5 \times 10^5$ ct/min and that for normal August rats being $9 \cdot 5 \times 10^4$ ct/min. However, the isotope uptake of lymph node cells from tumour bearing rats cultured without PHA was significantly higher than the uptake of lymph node cells from normal rats.

*MLC reaction between August and Wistar rat lymph node cells*

The time course of the two-way mixed lymphocyte reaction was examined by culturing together equal numbers ($10^6 + 10^6$) of August and Wistar rat lymph node cells and measuring the $^3$H-thymidine incorporation on Days 2–7. The response reached a peak of $3 \times 10^4$ ct/min/culture on Days 4 and 5 of culture, one day later than the PHA response (Fig. 4). The mean incorporation for 20 separate MLC reactions terminated on the 4th day of culture was $4 \cdot 6 \times 10^4$ ct/min/culture.

One-way mixed lymphocyte reactions were also examined. For this purpose lymph node cells from August and Wistar rats were treated with mitomycin C and various numbers of these cells were cultured for 4 days with $10^6$ untreated lymph node cells of the other strain.

FIG. 4.—Two-way MLC between $10^6$ LNC from normal August and normal Wistar rats (●——●). The control gives the sum of the incorporation by $10^6$ August and Wistar LNC cultured separately(■——■).

The response of Wistar rat lymph node cells to mitomycin C treated August rat lymph node cells was the same as the response of August rat lymph node cells to mitomycin C treated Wistar rat lymph node cells (Fig. 5). In both cases the peak response was obtained with $1–2 \times 10^6$ mitomycin C treated cells added to $10^6$ responder cells. Control cultures in which mitomycin C treated lymph node cells of either strain were cultured with syngeneic untreated lymph node cells did not show any evidence of stimulation.

FIG. 5.—One-way MLC between LNC from normal August and normal Wistar rats. August LNC were cultured with mitomycin C treated Wistar rat LNC (●———●) and Wistar rat LNC with mitomycin C treated August rat LNC (●- - - -●) Control cultures contained August (▲———▲) and Wistar (▲- - - -▲) rat LNC cultured with syngeneic mitomycin C treated LNC. All cultures were for 4 days and contained $10^6$ responder LNC.

One-way MLC reactions performed using irradiated (2000 rad) lymph node cells as the stimulating cells instead of mitomycin C treated cells produced essentially the same reaction.

*Stimulation of lymph node cells by irradiated tumour cells*

Irradiated tumour cells were cultured alone for 3–6 days and the $^3$H-thymidine uptake was determined (Fig. 6). On Day 3 there was still some isotope uptake (1800 ct/min/$10^6$ cells) but on Days 4, 5 and 6 this had fallen to background levels (150–500 ct/min/$10^6$ cells).

In preliminary experiments, tumour cells were also treated with mitomycin C (50 $\mu$g/ml for 30 min) to block DNA synthesis, but the background level of isotope incorporation by these cells was found to be more variable than for irradiated tumour cells.

Syngeneic and allogeneic lymph node cells from normal animals were incubated for 4 days with irradiated tumour cells. Each tube contained $10^6$ lymph node cells to which were added various numbers of tumour cells ranging from 0·25 to 2·0 × $10^6$/tube. It was found that the syngeneic lymph node cells incorporated

FIG. 6.—Time course of the response of syngeneic August rat LNC (a) and allogeneic Wistar rat LNC (b) to irradiated August tumour cells. (Tumour B.) (●———●) = $10^6$ LNC cultured alone, (▲———▲) = $10^6$ tumour cells cultured alone, (■———■) = mixed cell culture ($10^6$ LNC + $10^6$ tumour cells) and (○- - -○) = incorporation by mixed cell cultures corrected for tumour background.

FIG. 7.—Stimulation of $10^6$ normal August rat LNC by incubation with irradiated syngeneic tumour cells (tumour B) for 4 days. Each line shows the result of a separate experiment. The background incorporation by tumour cells alone has been subtracted from the value for mixed cell cultures.

FIG. 8.—Stimulation of $10^6$ normal Wistar rat LNC by incubation with irradiated August tumour cells (tumour B) for 4 days. Each line shows the result for a separate experiment. The background incorporation by tumour cells alone has been subtracted from the value for mixed cell cultures.

increased amounts of $^3$H-thymidine in the presence of tumour cells but only over a limited range of dilutions (0·5–1·0 × $10^6$ tumour cells/tube). The maximum response occurred with equal numbers of lymph node cells and tumour cells and represented a stimulation index of 4–8 (Fig. 7).

Allogeneic Wistar rat lymph node cells were also stimulated by the irradiated tumour cells. The response produced was greater than that produced by syngeneic August rat lymph node cells and occurred over a wider range of tumour cell dilutions, but again the maximum response was usually obtained with equal numbers of lymph node cells and tumour cells and represents a stimulation index of 10–40 (Fig. 8).

A similar experiment was performed using tumour P and the results obtained with both syngeneic and allogeneic LNC fell within the range of values obtained using tumour B.

In order to examine the time course of the response cultures were set up containing $10^6$ lymph node cells from August or Wistar rats and $10^6$ irradiated cells from tumour B. $^3$H-thymidine uptake was measured on Days 3–6 (Fig. 6). With syngeneic August rat lymph node cells, a significant response was found only on Days 4 and 5, whereas with allogeneic rat lymph node cells a response was found on all 4 days and was greatest on Days 4 and 5.

The effect of incubating irradiated tumour cells with serum before adding them to syngeneic and allogeneic lymph node cells was also examined. Irradiated tumour cells (tumour B) were incubated with serum from normal August rats, normal Wistar rats, tumour bearing August rats and Wistar rats which had

TABLE.—*The Effect of Serum Pretreatment of Irradiated Tumour Cells on the Response of Syngeneic August Rat Lymph Node Cells (LNC) and Allogeneic Wistar Rat LNC*

| | | | $^3$H-Thymidine ct/min/culture | | | |
|---|---|---|---|---|---|---|
| | | | Tumour + August LNC | | Tumour + Wistar LNC | |
| Tumour pretreatment | | Tumour only | Total count | Corrected for tumour background | Total count | Corrected for tumour background |
| MEM | | 224 | 1284 | 1060 | 3874 | 3650 |
| 10% Serum | Normal August | 281 | 602 | 321 | 6117 | 5836 |
| | Normal Wistar | 250 | 702 | 452 | 5351 | 5101 |
| | August tumour bearer | 203 | 609 | 406 | 6206 | 6002 |
| | Wistar anti-tumour | 214 | 464 | 250 | 2414 | 2200 |

August LNC only: 233
Wistar LNC only: 181

Tumour cells were incubated with 10% serum before being cultured with LNC. $10^6$ tumour cells were cultured with $10^6$ LNC for 4 days. $^3$H-thymidine uptake in mixed cell cultures is expressed as total counts per culture and as values corrected for background incorporation by tumour cells.

rejected a graft of tumour B. The treated tumour cells were washed and put up in culture with August or Wistar rat lymph node cells for 4 days and the $^3$H-thymidine uptake determined. As expected, pretreatment of the tumour cells with Wistar anti-tumour serum did reduce the response of both syngeneic and allogeneic lymph node cells compared with the response to MEM treated tumour cells (Table). However, it was found that pretreatment with serum from normal rats of either strain and serum from tumour bearing animals also reduced the response of syngeneic LNC, whereas the response of allogeneic lymph node cells was not reduced by these treatments—in fact it was somewhat higher.

Irradiated tumour cells pretreated with normal August serum, normal Wistar serum, Wistar anti-tumour serum, August tumour bearing rat serum or MEM were also cultured alone for 4 days and the $^3$H-thymidine incorporation was measured (Table). No significant differences were found between the levels of isotope uptake by cells treated with the various sera or MEM and the incorporation was similar to the levels obtained for untreated cells used in other experiments.

The present study did not involve an extensive examination of the response of lymph node cells from syngeneic and allogeneic rats presensitized to the tumour. However, in a small number of cases where lymph node cells were taken from syngeneic tumour bearing rats and allogeneic rats that had rejected a tumour graft, the responses produced were similar to those obtained with lymph node cells from normal syngeneic and allogeneic rats.

### DISCUSSION

The results obtained for lymph node cell stimulation by PHA and MLC are essentially the same as those reported by many workers using human and animal lymphocytes (Dutton, 1967; Ling, 1968). Very large responses are produced by PHA stimulation and MLC reactions and these are due to stimulation of a large number of cells. Bain *et al.* (1964) reported that up to 5% of the cells in a MLC respond while Bach and Hirschhorn (1964) suggest that a much higher percentage respond. PHA has been found to stimulate 30–80% of the cells in culture (Cowling, Quaglino and Davidson, 1963; Robbins, 1964; Dutton, 1967). On the other hand, antigenic stimulation of lymph node cells from immunized donors stimulated only 1–5% of the cells present (Dutton, 1961; Cowling *et al.*, 1963).

It is well established that lymphocytes from patients with tumours of the lymphoid system have depressed PHA responsiveness, but there are conflicting reports concerning lymphocytes from patients with non-lymphoid tumours; some workers have observed reduced responsiveness and others a normal response (Lauder and Bone, 1973). In the present study, the PHA response produced by LNC from rats bearing transplanted tumours was found to be normal; however, Gillette and Boone (1973) reported that spleen cells from animals with chemical or viral induced tumours showed a reduced response to PHA whereas lymph node cells responded normally.

In the present study, lymph node cells from normal August rats were stimulated when incubated with irradiated syngeneic tumour cells. Stimulation may be due to the recognition of antigenic differences between tumour cells and normal cells since the kinetics of the response are similar to the kinetics of the MLC reaction between histoincompatible rats and the reaction could be blocked by anti-tumour antibody. It is possible that irradiation of the tumour cells may have resulted in the production of antigens not present on unirradiated cells, but control experiments demonstrated that normal lymph node cells were not stimulated by irradiated syngeneic lymph node cells.

The response of August rat lymph node cells to syngeneic irradiated tumour cells is very similar to the responses obtained by other workers using blood lymphocytes and syngeneic tumour cells (irradiated or mitomycin C treated) from patients with solid tumours or leukaemias (Fridman and Kourilsky, 1969; Stjernswärd *et al.*, 1970; Vánky *et al.*, 1971*a*, *b*; Han and Wang, 1972; Gutterman *et al.*, 1973; Mavligit *et al.*, 1973). Stimulation of autologous lymphocytes has not been found in all the cancer patients tested. Gutterman *et al.* (1973) obtained stimulation with 24 out of 34 leukaemia patients, Fridman and Kourilsky (1969) 6 out of 10 leukaemia patients and Stjernswärd *et al.* (1970) only 3 out of 6 patients with renal carcinoma. These negative results suggest that lymphocytes from these patients were unable to respond to their own tumour cells. The negative results have not so far been correlated with the clinical condition of the patient.

Steel *et al.* (1973) have reported stimulation of lymphocytes by irradiated autologous lymphoblastoid cell lines derived from patients with infectious mononucleosis. They suggest that stimulation may have been due to antigens on the blast cells which are unmasked or acquired as the cells pass from a normal non-dividing state into one of active division. Cultured human tumour cells have also been shown to stimulate an *in vitro* blastogenic response by normal allogeneic lymphocytes (Anderson, McBride and Hersh, 1972).

It has been demonstrated that the mixed lymphocyte reaction can be blocked by treating the stimulator cells with an antiserum directed against them (Milton *et al.*, 1973; Nishihara and Fujii, 1973). In the present study, it was expected that treatment of the tumour cells with a Wistar anti-tumour serum would block the mixed lymphocyte–tumour cell reaction, and that this type of inhibition could be used as a test for anti-tumour antibody. Wistar anti-tumour serum did reduce the response of both syngeneic and allogeneic lymph node cells, but serum from normal rats of either strain and from tumour bearing animals also reduced the response of syngeneic lymph node cells.

In some human cases the lymphocyte blastogenic response to autologous tumour cells was blocked by adding serum from the same individual, though not all sera from tumour bearing patients have this property (Vánky *et al.*, 1971*b*, 1973; Gutterman *et al.*, 1973). A small percentage of normal human sera also produced some blocking. Thus, it appears that some human tumours provoke a blastogenic response and production of a blocking factor, others provoke only a blastogenic response and some do not provoke any response. In the rat system preincubation of the tumour cells with serum from normal August or Wistar rats produced the same blocking effect as preincubation with serum from tumour bearing rats. Titration experiments might show a quantitative difference between the level of blocking factor in sera from normal and tumour bearing rats but it seems more likely that the effect was produced by normal serum components. The nature of these factors remains to be determined but preliminary experiments suggest that they are not present in the IgG fraction of normal serum and that they are not heat labile. Several serum factors which can interfere with the stimulation of lymphocytes have been described by other workers and may be involved in the blocking effect. Nelson (1972) reported that normal mouse serum depressed the response of mouse spleen cells to PHA stimulation and MLC and that the factors responsible were not present in the IgG containing fraction of normal serum. $\alpha$ globulins prepared from normal human serum have been shown to interfere with stimulation of lymphocytes by PHA and specific antigens (Cooperband *et al.*, 1968). On the other hand, Currie and Bagshawe (1967) have proposed that tumour antigens are masked by sialomucin present on the surface of the tumour cells and this type of compound may be involved in the blocking effect of normal serum.

Preincubation of the irradiated tumour cells with MEM alone or with the various sera used in this experiment did not alter the thymidine incorporation of the tumour cells themselves. However, in later experiments employing the same tumour incubation with MEM increased the thymidine incorporation of the tumour cells and this effect was partly reversed by addition of 10% normal rat serum but not by tumour bearing serum. The results of these experiments will be reported elsewhere.

This work was carried out during the tenure by M. R. P. of a Medical Research Council studentship.

### REFERENCES

ANDERSON, R. J., MCBRIDE, C. M. & HERSH, E. M. (1972) Lymphocyte Blastogenic Responses to Cultured Allogeneic Tumor Cells *in vitro*. *Cancer Res.*, **32**, 988.

BACH, F. & HIRSCHHORN, K. (1964) Lymphocyte Interaction: A Potential Histocompatibility Test *in vitro*. *Science, N.Y.*, **143**, 813.

BAIN, B., VAS, M. R. & LOWENSTEIN, L. (1964) The Development of Large Immature Mononuclear Cells in Mixed Leukocyte Cultures. *Blood*, **23**, 108.

COOPERBAND, S. R., BONDEVIK, H., SCHMID, K. & MANNICK, J. A. (1968) Transformation of Human Lymphocytes: Inhibition by Homologous Alpha Globulin. *Science, N.Y.*, **159**, 1243.

COWLING, D. C., QUAGLINO, D. & DAVIDSON, E. (1963) Changes Induced by Tuberculin in Leucocyte Cultures. *Lancet*, ii, 1091.

CURRIE, G. A. & BAGSHAWE, K. D. (1967) The Masking of Antigens on Trophoblast and Cancer Cells. *Lancet*, i, 708.

DUTTON, R. W. (1961) Importance of Cell Division for Antibody Production in an *in vitro* System. *Nature, Lond.*, **192**, 462.

DUTTON, R. W. (1967) *In vitro* Studies of Immunological Responses of Lymphoid Cells. *Adv. Immunol.*, **6**, 253.

FRIDMAN, W. H. & KOURILSKY, F. M. (1969) Stimulation of Lymphocytes by Autologous Leukaemic Cells in Acute Leukaemia. *Nature, Lond.*, **224**, 277.

GILLETTE, R. W. & BOONE, C. W. (1973) Changes in Phytohemagglutinin Response due to the Presence of Tumors. *J. natn. Cancer Inst.*, **50**, 1391.

GUTTERMAN, J. U., ROSSEN, R. D., BUTLER, W. T., MCCREDIE, K. B., BODEY, G. P., FREIREICH, E. J. & HERSCH, E. M. (1973) Immunoglobulin on Tumor Cells and Tumor-induced Lymphocyte Blastogenesis in Human Acute Leukemia. *New Engl. J. med. Sci.*, **288**, 169.

HAN, T. & WANG, J. (1972) Antigenic Disparity Between Leukaemic Lymphoblasts and Normal Lymphocytes in Identical Twins. *Clin. & exp. Immunol.*, **12**, 171.

HARRIS, R., (1973) Leukaemia Antigens and Immunity. *Nature, Lond.*, **241**, 95.

HIRSCHHORN, K., BACK, F., KOLODNY, R. L., FIRSCHEIN, I. L. & HASHEM, N. (1963) Immune Response and Mitosis of Human Peripheral Blood Lymphocytes *in vitro*. *Science, N.Y.*, **142**, 1185.

LAUDER, I. & BONE, G. (1963) Lymphocyte Transformation in Large Bowel Cancer. *Br. J. Cancer*, **27**, 409.

LING, N. R. (1968) *Lymphocyte Stimulation*. North-Holland Publishing Co.: Amsterdam

MAVLIGIT, G. M., HERSH, E. M. & MCBRIDE, C. M. (1973) Lymphocyte Blastogenic Responses to Autochthonous Viable and Nonviable Human Tumor Cells. *J. natn. Cancer Inst.*, **51**, 337.

MILTON, J. B., MOMBRAY, J. F., RUSZKIEWICZ, M. & CARPENTER, C. B. (1973) Antiimmunogenic

Effect of Specific Antibody on the Mixed Lymphocyte Reaction. *Transplantation*, **15**, 579.

NELSON, D. S. (1972) Mouse Serum Factor Depressing Lymphocyte Transformation. *Experimentia*, **28**, 1227.

NISHIHARA, T. & FUJII, G. (1973) Effect of Humoral Antibodies on the Mixed Lymphocyte Culture Reaction in Rats. *Jap. J. exp. Med.*, **42**, 495.

ROBBINS, J. H. (1964) Tissue Culture Studies of the Human Lymphocyte. *Science, N.Y.*, **146**, 1648.

STEEL, C. M., HARDY, D. A., LING, N. R., DICK, H. M., MACKINTOSH, P. & CRICHTON, W. B. (1973) The Interaction of Normal Lymphocytes and Cells from Lymphoid Cell Lines. *Immunology*, **24**, 177.

STJERNSWÄRD, J., ALMGARD, L. E., FRANZEN, S., VON SCHREEB, T. & WADSTROM, L. B. (1970) Tumour Distinctive Cellular Immunity to Renal Carcinoma. *Clin. & exp. Immunol.*, **6**, 963.

VÁNKY, F., STJERNSWÄRD, J. & NILSONNE, U. (1971*a*) Cellular Immunity to Human Sarcoma. *J. natn. Cancer Inst.*, **46**, 1145.

VÄNKY, F., STJERNSWÄRD, J., KLEIN, G. & NILSONNE, U. (1971*b*) Serum-mediated Inhibition of Lymphocyte Stimulation by Autochthonous Human Tumors. *J. natn. Cancer Inst.*, **47**, 95.

VÁNKY, F., STJERNSWÄRD, J. KLEIN, G., STEINER, L. & LINDBERG, L. (1973) Tumor-associated Specificity of Serum-mediated Inhibition of Lymphocyte Stimulation by Autochthonous Human Tumors. *J. natn. Cancer Inst.*, **51**, 25.